# Causes of Mortality and Disease in Rabbits and Hares: A Retrospective Study

**DOI:** 10.3390/ani10010158

**Published:** 2020-01-17

**Authors:** José Espinosa, M. Carmen Ferreras, Julio Benavides, Nerea Cuesta, Claudia Pérez, M. José García Iglesias, J. Francisco García Marín, Valentín Pérez

**Affiliations:** 1 Departamento de Sanidad Animal, Facultad de Veterinaria, Campus de Vegazana s/n, Universidad de León, 24071 León, Spain; mcfere@unileon.es (M.C.F.); julio.benavides@csic.es (J.B.); ncueg@unileon.es (N.C.); cperm@unileon.es (C.P.); mjgari@unileon.es (M.J.G.I.); jfgarm@unileon.es (J.F.G.M.); valentin.perez@unileon.es (V.P.); 2 Departamento de Sanidad Animal, Instituto de Ganadería de Montaña (CSIC-Universidad de León), Finca Marzanas, Grulleros, 24346 León, Spain

**Keywords:** disease, condition, mortality, necropsy, rabbit, risk factor, pathology, hare, histopathology, microbiology

## Abstract

**Simple Summary:**

Domestic and wild lagomorphs, especially rabbits and hares, are important from an economic, ecological and public health point of view. Both rabbits and hares are susceptible to a wide variety of pathological disorders, so that the knowledge of the different risk factors, causes of death or disease and prevalence rates is relevant from a health, economic and welfare perspective. Despite the accumulation of information on lagomorph medicine and pathology, comprehensive published compilations of diagnostic cases in rabbits and hares are scarce. Between 2000 and 2018, 325 lagomorphs (rabbits and hares) from northern Spain were necropsied. A wide variety of conditions were identified. The health problems most frequently diagnosed were related to parasitic conditions (*n* = 65; 24.34%), bacterial diseases (*n* = 56; 20.97%), nutritional and metabolic disorders (*n* = 48; 17.97%), viral infections (*n* = 31; 11.61%), miscellaneous causes (*n* = 31; 11.61%), neoplasms (*n* = 12; 4.49%), toxicoses (*n* = 11; 4.11%), trauma-related injuries (*n* = 9; 3.37%) and finally, congenital diseases (*n* = 4; 1.49%). The species, sex, age and time of the year were predisposing factors in many of the conditions identified. The frequency of presentation and main pathological findings of these disorders were consistent with the most important lagomorph diseases reported in other European countries and other referenced studies.

**Abstract:**

In this study we determined the causes of mortality and disease in a total of 325 lagomorphs (rabbits and hares) in northern Spain between 2000 and 2018. Risk factors such as the species, age, sex, time of year and origin were also considered. Clinical signs, gross and histopathological findings and ancillary test results were the basis for the final diagnoses that were reviewed to classify and identify the different disorders. A total of 26 different conditions were identified. A single cause of death or illness was detected in 267 animals. They were grouped into parasitic conditions (*n*= 65; 24.34%) represented by encephalitozoonosis, hepatic coccidiosis, hepatoperitoneal cysticercosis, intestinal coccidiosis, parasitic gastritis and cutaneous ectoparasitosis; bacterial diseases (*n* = 56; 20.97%) including pseudotuberculosis, blue breast, skin abscesses, tularemia, pneumonic pasteurellosis and staphylococcal infections; nutritional and metabolic diseases (*n* = 48; 17.97%) with epizootic rabbit enteropathy, hepatic steatosis and pregnancy toxemia as prominent diseases; viral infections (*n*= 31; 11.61%) comprising rabbit hemorrhagic disease and myxomatosis and miscellaneous causes (*n* = 31; 11.61%) where rabbit enteritis complex, renal conditions (nephrosis), heat stroke, and arterial bone metaplasia were included; neoplasms (*n* = 12; 4.49%) represented by uterine adenocarcinoma, mammary adenocarcinoma, cutaneous fibroma, intestinal lymphoma and hepatic cholangiocarcinoma; toxicoses (*n* = 11; 4.11%); trauma-related injuries (*n* = 9; 3.37%) and finally congenital diseases (*n* = 4; 1.49%). In 58 animals of the study, some of these conditions were presented jointly. We discuss the detection frequency, possible causes or associated factors of the different pathologies as well as the importance of the different variables considered.

## 1. Introduction

The species of the family Leporidae (rabbits and hares), from the order Lagomorpha, include both individuals that have been domesticated as companion and/or production animals used in the food or fur industry as well as in biomedical research [1], and those wild animals that are part of natural ecosystems. Diseases have been closely linked to lagomorphs. In the raising of rabbits in rural areas and in wildlife lagomorphs, disease has always been present [2] (pp. 481–498). However, the social and economic changes of the past few decades have caused lagomorph medicine to evolve significantly and to have become more important than ever, due to increased productivity and economic needs [3] of the farm industry.

The health of industrially farmed rabbit populations is of great relevance from a productive and financial point of view [4]. Many animals are culled from farms as a result of disease or low productivity. Despite the significant progress experienced in the field of genetics, feeding and management, the increase in productivity, the high selection of breeds and their crosses make the onset of diseases unavoidable [5,6]. This implies an additional expense in a sector where, today, production costs outweigh benefits. In addition, the increasing demanded for ecological cuniculture, where the use of synthetic drugs is not allowed, makes the knowledge of both the associated different disorders and proper health management key factors in this sector. In the case of wildlife lagomorphs, their importance lies in their ecological value as species within the food chain, their economic value for hunting, and their being a reservoir of zoonoses and economically important diseases [7,8]. In this sense, the evaluation of dead animals within epidemiological surveillance plans is crucial for the evaluation of the health status of wild populations. At the same time, the extraordinary boom that pet rabbits have had recently in European and North American societies emphasizes the importance of knowledge of the different associated diseases from a clinical and public health (zoonosis) point of view.

In most cases, in both domestic and wild species, the study of the disease is approached from the perspective of the community (without belittling valuable individuals, such of pet) and integrated into the medicine of production and/or conservation. This makes the mortality risk factors, causes of death and prevalence rates of the different pathological conditions variables of interest from a health and welfare perspective [9] (pp. 237–245). The greater our knowledge is, the better we can diagnose rabbit and hare diseases, enhance their prevention, and choose the appropriate control method for a specific population [10].

Despite the accumulation of information on lagomorph medicine and pathology, comprehensive published compilations on lagomorph diagnostic cases are relatively scarce. The purpose of this study is to report a retrospective evaluation of diagnostic cases found in lagomorphs (rabbits and hares) received over an 18-year period at the Department of Animal Health of the Faculty of Veterinary Sciences of the University of León, a region in the Northwest of Spain

Where there is a significant lagomorph population, both in wildlife and farms. Our aims were both to determine the prevalence of the diagnosed diseases considering risk factors such as species, age, sex, time of year and origin, and to characterize those diseases or illness through their gross and histopathological features after necropsy. Our challenge was to contribute to the knowledge and prevention framework of lagomorph disease.

## 2. Materials and Methods 

### 2.1. Animals

From 19 January 2000 to 27 November 2018, we obtained the data of our study from a total of 325 necropsies performed on diseased and dead lagomorphs referred to the Diagnostic Service of the Department of Animal Health of the Faculty of Veterinary Sciences (University of León, Spain). Whenever possible, the referring owners or veterinarians of each animal submitted to the laboratory provided a brief clinical history (duration and type of clinical signs, number of animals affected, etc.), the sex, age and exact date of death of the animal. Based on this information, the animals were classified according to the species (hares or rabbits), sex, age category (young; less than 7 months of age, adults; over 8 months) [11] (pp. 189–249), origin (farm; meat production animals raised in a traditional way and/or by industrial breeding farms; wild and pets) and time of year in which the reception of the animal was registered (spring–summer: 20 March–20 September; autumn–winter: 21 September–19 March). The vast majority of farm and pet rabbits belonged to the New Zealand breed. The distribution of animals according to the categories described is shown in Table 1. All data were recorded before performing the regulated necropsy of each animal included in the study.

Animal Care and Use Committee approval was not obtained for this study because data were gathered from animals raised under commercial conditions and wild animals were recovered by the Environment Agents of the Junta de Castilla y León, regional government, fulfilling European, Spanish, and regional recommendations and laws on animal welfare and wildlife management.

### 2.2. Necropsy, Histopathological and Microbiological Diagnostic Workup 

The 325 animals included in the study were submitted to the Diagnostic Laboratory mostly within 24 h of death. Many of the specimens that arrived at the laboratory from commercial rabbit farms showed different degrees of autolysis because dead rabbits were removed daily from the farm facilities to the area for rendering (refrigerator and freezer) as a biosecurity measure [12] (pp. 1–11), [13] (pp. 337–369). In this study, only carcasses in good condition were included to accurately describe the gross and histopathological findings after necropsy and identify the exact cause of death or illness. The necropsy protocols used were those described by Corpa (2009) [14]. Gross examination was performed on all animals and organs following a standard protocol. A systematic and complete sampling of tissues from each animal was carried out for histological analysis. All samples were fixed in 10% neutral buffered formalin for at least 48–72 h and stored until histological processing. In turn, when suspected gross lesions were observed, tissue samples were collected in sterile tubes and stored at −20 °C until microbiological analysis took place. 

For histopathological examination, tissue samples were briefly washed with 10% phosphate buffered saline (PBS) solution, dehydrated in alcohol and embedded in paraffin wax (EI LEICA TP1020 Automatic Tissue Processor^®^, Barcelona, Spain). Serial 5 μm sections from all specimens were mounted on glass slides (Super-Frost, Menzel-Gläser, Braunschweig, Germany), stained with hematoxylin and eosin (HE) and examined by light microscopy. Additional stains were performed as needed and most commonly included Gram, periodic acid-Schiff, Giemsa and Ziehl-Neelsen stains. Lesions were examined by the authors, who included three fully qualified European College of Veterinary Pathologists (ECVP) diplomates (V.P, J.F.G.M and N.C).

For microbiological studies, after flame the tissue samples, one gram of each tissue and/or pathological material was placed in a sterile airtight bag with 3 mL of sterile 10% PBS and homogenized for 5 min (Stomacher 80 Biomaster^®^, Lardero, Spain). Samples were cultured on Blood and MacConkey agar plates (supplemented with streptomycin when necessary; bioMérieux, Madrid, Spain) and incubated aerobically and/or anaerobically at 37 °C for 24–48 h. Direct observation of the bacterial colonies was carried out after specific staining (Gram). Bacterial strains were identified by analyzing the macro and micromorphological characteristics, physiological, enzymatic and/or biochemical tests of the different microorganisms with the help of API gallery systems (Biomerieux^®^)and, according to the type of bacterial agent, with visual reading and analysis in API software Web version 4.0 (Biomerieux^®^) or automated biochemical tests by Vitek^®^ System Cards (BioMérieux^®^). Between 2014 and 2018 all isolates were analyzed in a Bruker DaltonikUltrafleXtreme MALDI TOF/TOF (desorption/ionization time-of-flight mass spectrometry) system, which obtained one spectrum per sample used to assess the suitability of this spectrometric approach for identification at the species level. Each spectrum was acquired using FlexControl software (Version 3.4) in automatic mode in a random sampling pattern. The identification of all the clinical isolates in this study was performed by MALDI Biotyper Real Time Classification software. The reliability of the identification was evaluated from the log (score) values, calculated with the MALDI Biotyper software mentioned above.

Diagnosis of viral, parasitic and toxic diseases was based on the observed clinical-epidemiological and histopathological features. Identification of parasitic agents was carried out according to their morphological characteristics.

### 2.3. Statistical Analyses

The different processes diagnosed were expressed as percentages with respect to the total number of conditions detected in each group. Non-parametric statistical methods were used to compare between groups. Specifically, the Pearson Chi-squared with Yates continuity correction test was used to contrast the relationship between the detection frequency of each condition with the different variables considered (species, age category, sex, time of year and origin). Where necessary, in a second step, Pearson Chi-squared with Bonferroni correction post-hoc comparison was performed to determine the differences among the groups analyzed [15] (pp. 243–355). *p*-values < 0.05 were considered to be statistically significant.

All statistical analyses were performed with the R software version 3.5.3 [16].

## 3. Results

Final diagnoses were achieved after considering the clinical signs, gross and histopathological findings, and ancillary test results. They were grouped into two wide categories so that distinction was made between diseases or conditions diagnosed as a single cause of death or illness, or cases in which more than one disease/condition was diagnosed per animal.

### 3.1. Conditions Diagnosed as a Single Cause of Death or Illness

Diseases or conditions diagnosed as the only cause of death or disease were recognized in 267 (82.15%) of the 365 animals included in the study. A total of 25 different disorders were identified (see Table 2). The most affected lagomorphs of the study were rabbits (*n* = 238; 89.13%), young (*n* = 152; 56.92%), females (*n* = 185; 69.28%) submitted during the autumn–winter period (*n* = 171; 64.04%) and individuals that came from a farm origin (67.04%; *p* < 0.01). In this group of animals, the most frequently health problems were the following: parasitic conditions (*n* = 65; 24.34%), bacterial diseases (*n* = 56; 20.97%), nutritional and metabolic diseases (*n* = 48; 17.97%), viral infections (*n* = 31; 11.61%), miscellaneous causes (n = 31; 11.61%), neoplasms (*n* = 12; 4.49%), toxicoses (*n* = 11; 4.11%), trauma-related injuries (*n* = 9; 3.37%) and finally, congenital diseases (*n* = 4; 1.49%).

#### 3.1.1. Parasitic Diseases

*Encephalitozoonosis* (*n* = 25; 38.66%) was the disease most frequently diagnosed, followed by hepatic coccidiosis (*n* = 13; 20%), hepatoperitoneal cysticercosis (*n* = 9; 13.84%), intestinal coccidiosis (*n* = 7; 10.76%), parasitic gastritis (*n* = 7; 10.76%) and cutaneous ectoparasitosis (*n* = 4; 6.15%). The rabbit was the species most affected by parasitic diseases (90.66%; *p* < 0.001) with an industrial or farm origin (84.61%; *p* < 0.001). These diseases were more prevalent in young individuals (72.31%; *p* < 0.001) while no differences were found in the frequency of detection between sexes (*p* = 0.321). The frequency of diagnosis of parasitic diseases was significantly higher in the animals submitted during the autumn–winter period (72.30%; *p* < 0.001).

*Encephalitozoonosis.* All affected animals were rabbits, mainly farm (64%; *p* < 0.01), young (64%; *p* < 0.01) and female animals remitted during the autumn–winter period (52%; *p* = 0.682). The general clinical signs were apathy, mild retarded growth and weight loss. Six animals presented muscular weakness, ataxia and paralysis of the hind limbs. No animals with ocular clinical signs were reported. The only gross lesion observed was the presence of focal, irregular and depressed pale areas on the renal cortical surface (Figure S1) as well as ulcers in the gastric mucosa. Histologically, the kidneys showed a multifocal granulomatous interstitial nephritis with associated tubular ectasia showing a few degenerated tubular cells, as well as congestion. At the central nervous central system (CNS) level, there was a multifocal, nodular, non-suppurative and granulomatous meningoencephalomyelitis with astrogliosis and perivascular lymphocytic cuffs. No lesions were detected in other organs. With Gram and Giemsa stains, the presence of spores and pseudocysts consistent with *Encephalitozoon cuniculi* was visualized inside the cells of the phagocytic mononuclear system of the inflammatory infiltrates in the CNS and into the vascular endothelial and renal tubular epithelial cells of the kidneys. 

*Hepatic coccidiosis.* All affected animals were farm rabbits, mainly young (84.61%; *p* < 0.001), with no significant differences in the detection frequency between sexes (*p* = 0.203) or times of the year considered in the study (*p* = 0.509). The clinical signs were anorexia, weight loss and mild diarrhea. Gross lesions included hepatomegaly with multifocal to coalescing irregularly shaped, raised, yellow-white nodules or cords in the liver that, at cross section, were consistent with dilated bile channels (Figure S2). Histologically, there was bile duct ectasia with hyperplasia characterized by papillary projections composed of reactive epithelial cells in which gametocytes and oocysts compatible with *Eimeria stiedae* were seen in their cytoplasm. They were also freely present in the ductal lumina. 

*Hepatoperitoneal cysticercosis.* This condition was detected mainly in wild hares (66.66%; *p* < 0.01) and adult females (77.80%; *p* < 0.01) remitted during the autumn–winter period (77.89%; *p* < 0.01). The only clinical sign was a low body condition. Gross lesions included the presence of parasitic cysts in liver surface, peritoneal, diaphragmatic and intestinal serosa (Figure S3). Histologically, there was a multifocal to disseminated interstitial chronic hepatitis composed of a granulomatous inflammation and fibrous reaction associated with cysts of *Cysticercus pisiformis*.

*Intestinal coccidiosis*. All affected animals were young farm rabbits submitted during the autumn–winter season. No differences were found between sexes (*p* > 0.601). Affected animals showed delayed growth, anorexia, abdominal pain, prostration and a mucous greenish diarrhea. Gross pathological findings included dilated intestinal loops observed mainly in the jejunum and ileum areas and sometimes in the caecum, along with the presence of small whitish foci in the intestinal serosa and reactive mesenteric lymph nodes. Histologically, there was destruction and necrosis of enterocytes and crypts, atrophy or villi and marked heterophilic infiltration. Giant schizonts and gametocytes consistent with *Eimeria* spp. could be seen in the lamina propria of the small intestine and in the epithelial cells covering the villi.

*Parasitic gastritis*. All animals with this condition were wild adult male rabbits remitted during the autumn–winter period. No obvious clinical signs were seen. Gross lesions included focal ulcerations, small foci of hemorrhages and a prominent mucous exudate in the gastric mucosa (Figure S4). Histologically, there was a catarrhal gastritis with a diffuse inflammatory infiltrate composed of lymphocytes, macrophages and eosinophils together with foci of fibrosis. Coiled larvae were detected in the lumen of the glands of the gastric fundus and adult worms were identified in the mucous exudate, with the head buried into the stomach grooves. They were morphologically consistent with *Graphidium* spp.

*Cutaneous ectoparasitosis*. Ectoparasite infestation was observed in adult female farm rabbits, submitted mainly during the spring–summer period (75%; *p* < 0.01). The animals were reported to have intense pruritus and marked thinness. Gross changes included crusty skin lesions in ears, head and distal parts of the limbs, with hyperemia and edema. Histologically, there was severe proliferative otitis and dermatitis with evident serocellular crusts. The epidermis was acanthotic with marked parakeratotic hyperkeratosis where mites and larvae could be seen embedded within adherent and exfoliated keratin, consistent with psoroptic mange.

#### 3.1.2. Bacterial Diseases

Six different bacterial conditions were identified with no differences in the detection frequency among them: pseudotuberculosis (*n* = 10; 17.85%), blue breast (*n* = 10; 17.85%), skin abscesses (*n* = 10; 17.85%), tularemia (*n* = 9; 16.07%), pneumonic pasteurellosis (*n* = 9; 16.07%) and finally staphylococcal infections (*n* = 8; 14.28%). The most affected species were rabbits (73.21%; *p* < 0.001) of industrial origin (67.85%; *p* < 0.001), adults (58.95%; *p* < 0.05) and females (85.71%; *p* < 0.001), received mainly during the autumn–winter period (71.42%; *p* < 0.001).

*Pseudotuberculosis.* This disease was most frequently diagnosed in wild specimens (80%; *p* < 0.001) and adult females (90%; *p* < 0.001) submitted during the autumn-winter period (80%; *p* < 0.001). No differences were found in the detection frequency between species (*p* = 0.067). Reported clinical signs were mild ataxia, anorexia and weight loss. Gross lesions included multiple white-yellowish necrotic miliary foci in liver, spleen, mesenteric lymph nodes, sacculus rotundus and cecal appendix, as well as generalized congestion (Figure S5). Histologically, these foci corresponded to multifocal to coalescing areas of lytic necrosis with neutrophils (microabscesses) and areas of granulomatous inflammation surrounding large florid colonies of amphophilic coccobacilli. The bacteriological culture confirmed the presence of *Yersinia enterocolitica*.

*Blue breast.* All affected animals were farm rabbits, female, mainly adult animals (90%; *p* < 0.001) remitted in the autumn–winter period (70%; *p* < 0.01). Clinically, the animals showed weight loss and breast-feeding rejection. Grossly, there was diffuse breast swelling with the skin overlying the mammary glands showed a red to dark blue discoloration and the presence of serous to purulent exudates. Histologically, there were areas of suppurative inflammation and necrosis of the mammary gland as well as embolization of coccoid bacterial colonies in vessels. *Staphylococcus aureus*, *Pasteurella multocida* and *Streptococcus* spp. were isolated from the lesions.

*Skin abscesses.* Animals with this condition were farm rabbits, mainly young (80%; *p* < 0.001), female (70%; *p* < 0.001), submitted during the autumn–winter period. They showed cachexia. Gross lesions included subcutaneous masses in the submandibular, neck, esternal and interscapular areas. Histologically, there were well-circumscribed abscesses containing numerous degenerated neutrophils. No abscesses were detected in other organic regions. *Pasteurella* spp. and *Staphylococcus* spp. were isolated from the damaged areas.

*Tularemia.* This condition was diagnosed in adult wild hares, mainly females (88.88%; *p* < 0.001) during the spring-summer period (88.88%; *p* < 0.001). Most of the animals were found dead but slow movements and nervous symptoms were seen prior to death. The animals showed a high degree of parasitism by ticks, hepato-, spleno- and generalized lymphadenomegaly with the presence of military small white necrotic foci (Figure S6), visceral congestion, ascites and hydrothorax. Histologically, affected organs contained multifocal to coalescing areas of lytic necrosis associated with pyogranulomatous inflammation and vasculitis. Bacteriologic culture confirmed the isolation of *Francisella tularensis*.

*Pneumonic pasteurellosis.* This condition was diagnosed in rabbits, mainly young females (88.88%; *p* < 0.001), with a farm origin (88.88%; *p* < 0.001), submitted during the autumn–winter period (88.88%; *p* < 0.001). The animals showed depression, anorexia, respiratory distress with mucopurulent rhinitis (snuffles) and blepharoconjunctivitis. Gross findings included serosanguineous secretion in the nasal cavity and respiratory airways, subcutaneous petechiae in the neck and thoracic area, fibrinopurulent pleuritis and pulmonary congestion (Figure S7). Histologically, there was an exudative bronchopneumonia with necrosis and heterophilic inflammation, disseminated intravascular coagulation (DIC) and variable fibrosis, and occasionally, multifocal non-purulent myocarditis. The diagnosis confirmed the infection by *P.multocida*, *Bordetella* spp. and in two animals, additionally *Staphylococcus* spp.

*Staphylococcal septicemia*. This condition was diagnosed in farm rabbits, mostly adults (62.5%; *p* = 0.012), females (75%; *p* < 0.001), remitted during the autumn–winter period (87.5%; *p* < 0.001). Clinically, the animals showed progressive loss of weight and cachexia. Gross lesions included the presence of generalized abscesses in the lung, liver, kidney, spleen, heart and skeletal muscle (Figure S8). Histologically, there was a multifocal fibrinosuppurative myocarditis, hepatitis, splenitis, nephritis and pneumonia. In addition, there was widespread hyalinization and segmental necrosis of muscle fibers and septic thromboarteritis, and occasionally, multifocal purulent encephalitis. The most commonly isolated agent was *S. aureus*.

#### 3.1.3. Nutritional and Metabolic Diseases

Within this disease group, three different conditions were identified. Epizootic rabbit enteropathy was the most frequently diagnosed (*n* = 27; 56.25%), followed by hepatic steatosis (*n* = 16; 33.33%) and pregnancy toxemia (*n* = 5; 10.41%). In general, all affected animals were farm rabbits, mostly young (68.75%, *p* < 0.01), and females (85.41%; *p* < 0.001) submitted during the autumn–winter period (79.16%; *p* < 0.001).

*Epizootic rabbit enteropathy*. This disease was diagnosed in farm rabbits, mainly young animals (77.77%; *p* < 0.001) and females (81.48%; *p* < 0.001) remitted during the autumn–winter period (70.37%; *p* < 0.001). Affected individuals showed anorexia, depression, mucous yellowish diarrhea, icterus and mild weight loss. Gross findings included distension of the stomach, distal segment of the ileum and proximal colon and caecum that showed impaction of the content and abundant translucent and gelatinous mucus (Figure S9). In some animals, small hemorrhages in the thymus and trachea could be observed. Histologically, the animals had extensive goblet cell hyperplasia and enterocyte vacuolation with mild epithelial loss and villous atrophy and an inflammatory infiltrate composed mainly of mononuclear cells. Periportal hepatic necrotic foci were observed in a few cases.

*Hepatic steatosis*. The affected animals were all farm rabbits, mainly young (75%; *p* < 0.001), and females, submitted during the autumn–winter period (87.5%; *p* < 0.001). The animals showed progressive weight loss, anorexia and depression. Gross lesions included hepatomegaly, hepatic discoloration and mild ascites. Histologically, there was fatty degeneration of hepatocytes with a panlobulillar distribution, and mononuclear inflammatory cell infiltrates around the centrolobular vein and portal spaces. 

*Pregnancy toxemia*. All affected animals were adult rabbit breeding does, remitted in the autumn–winter period. They were reported to show apathy, constipation, prostration, respiratory distress and lack of appetite. All the animals were obese, in advanced pregnancy and showed intense liver discoloration. Histologically, the liver lesions were similar to those described in hepatic steatosis. In addition, pulmonary congestion and alveolar edema, together with areas of vacuolation in the cerebral cortex were observed. No lesions were detected in the fetuses.

#### 3.1.4. Viral Diseases

Two viral conditions were detected in the studied population. The predominant disease was rabbit hemorrhagic disease (*n* = 22; 70.96%) followed by myxomatosis (*n* = 9; 29.03%). In general, the affected species was the rabbit, predominantly young animals (64.51%; *p* < 0.05), females (61.29%; *p* < 0.05), both with a farm and wild origin (*p* = 0.192) and submitted mainly during the spring -summer period (58.06%; *p* = 0.021).

*Rabbit hemorrhagic disease (RHD)*. Most affected animals were young rabbits (63.63%; *p* < 0.05), females (59.09%; *p* < 0.05) and wild (59.09%; *p* < 0.05) submitted during the spring–summer season (72.72%; *p* < 0.001). Diseased animals were found dead with a serosanguinous discharge in the nose and mouth. Gross lesions included serosal, pulmonary and perineal hemorrhages, hepato- and splenomegaly, with liver discoloration, and pulmonary congestion and edema (Figure S10). Histologically, there was severe diffuse hepatic single cell necrosis with heterophilic infiltration, pulmonary edema, hemorrhages and lymphocytolysis. There were fibrin thrombi in the capillaries and multisystemic DIC. 

*Myxomatosis*. This disease mainly affected young female rabbits (66.66%; *p* < 0.01), and farm animals during the autumn-winter period (77.77%; *p* < 0.001). Clinically, they showed blindness and progressive cachexia. Necropsy findings included pseudo-tumors along the face and anogenital region with severe edema and rhinitis and serous blepharoconjunctivitis (Figure S11). Subcutaneous masses were composed of undifferentiated mesenchymal cells within abundant myxomatous stroma. The overlying epidermis was hyperplastic with ballooning degeneration of keratinocytes of stratum spinosum and eosinophilic, large, intracytoplasmic inclusions in the epithelial cells. Lymphoid depletion and necrosis were present in lymph nodes and spleen. There was also moderate necrosis of pneumocytes and hepatocytes.

#### 3.1.5. Miscellaneous Causes

In this category, those conditions of diverse etiologies that could not be classified within the other established groups were included. These were the rabbit enteritis complex (*n* = 17; 54.83%); renal conditions (nephrosis; *n* = 6; 19.35%), acute shock (*n* = 5; 16.12%) and arterial bone metaplasia (*n* = 4; 12.90%).

*Rabbit enteritis complex*. This condition mainly affected young rabbits (88.23%; *p* < 0.001), females (58.82%; *p* < 0.05) and farm animals (76.47%; *p* < 0.001) during the autumn–winter period. The animals showed yellowish watery diarrhea and weight loss. Necropsy findings included severe intestinal dilation and mesenteric lymphadenomegaly (Figure S12). The histological lesion consisted of severe cecal and colonic mural edema with diffuse serosal hemorrhages in the cecum, and desquamation of epithelial cells with the presence of numerous bacterial colonies and gametocytes in the epithelial cells of the villi consistent with *Eimeria* spp. Additionally, these animals presented multifocal hepatic degeneration and DIC. The bacterial culture confirmed the presence of *Escherichia coli* and *Clostridium* spp.

*Nephrosis*. This degenerative renal condition was mostly detected in young rabbits from a farm origin (83.33%; *p* < 0.001), mainly females (66.66%; *p* < 0.01) submitted during the autumn–winter period. The animals presented with anorexia and prostration. No apparent gross lesions were observed. Histologically, there was cytoplasmic swelling and eosinophilia of the proximal tubular cells, nuclear kariorrhexis and the presence of proteinaceous material in the tubular lumen, as well as multifocal mineralization of the renal tubular epithelial cells and glomerular and tubular basement membranes.

*Acute shock*. Acute shock caused by heat stroke always affected young male pet rabbits during the spring–summer period. Animals were found dead and without showing previous clinical signs. Gross and histopathological lesions included mild hepatic discoloration, congestion and pulmonary edema, intestinal congestion and petechiae in the thymus and trachea. No other lesions compatible with other processes were identified and the microbiological cultures of tissues did not offer any significant result.

*Arterial bone metaplasia*. This condition was diagnosed in adult rabbits during the spring-summer period, mostly in females (75%; *p* < 0.001) and both farm and pets (*p* = 0.219). The animals showed anorexia and wasting. At necropsy, the aortic, pulmonary and carotid arteries appeared hardened, as rigid tubes with an irregular intimal surface. Histologically, there was calcification of elastic fibers of tunica media, foci of cartilaginous and bone metaplasia and bone marrow formation (Figure S13).

#### 3.1.6. Neoplasms

Tumors were diagnosed in adult rabbits, mainly females (75%; *p* < 0.001) and pet rabbits (91.66%; *p* < 0.001). The most frequently found neoplasm was uterine adenocarcinoma (*n* = 6; 50%) (Figure S14) followed by mammary adenocarcinoma (*n* = 3; 25%), cutaneous fibroma (*n* = 1; 8.33%), intestinal lymphoma (*n* = 1; 8.33%) and hepatic cholangiocarcinoma (*n* = 1; 8.33%). The common clinical sign was the presence of a low body condition. In the uterine adenocarcinoma, histological findings included acinar or tubular structures lined by cuboidal neoplastic epithelial cells supported by a collagenous fibrovascular stroma, as well as cystic endometrial hyperplasia and focal adenomyosis. Mammary adenocarcinoma was seen as a well-defined mass that histologically showed a tubulo-papillary growth of epithelial mammary cells, without vascular invasion. Cutaneous fibroma was composed of a proliferation of well differentiated fusiform cells with low mitotic index and low grade of anisocytosis and anisokaryosis. Hepatic cholangiocarcinoma consisted of polygonal epithelial cells with marked pleomorphism arranged in cords and tubules, with frequent mitoses. Finally, the intestinal lymphoma was a mass that locally invaded the small intestine mucosa, composed of lymphoid cells with marked anisocytosis and anisokaryosis and high mitotic index.

#### 3.1.7. Toxicoses

Toxic-related lesions were identified only in female farm rabbits, mainly in young animals during the autumn–winter period (72.72%; *p* < 0.001). They showed anorexia, mild diarrhea, dyspnea, ataxia, muscular atrophy and recumbency. Gross lesions included a mild hydropericardium, hydrothorax and myocardial and skeletal muscle white discoloration (Figure S15). The only histological changes were multifocal monophasic reactions consistent with severe hyaline degeneration and necrosis of skeletal muscle fibers and cardiac muscle with the presence of hypercontraction bands and mild histiocytic infiltration. 

#### 3.1.8. Traumatisms

Trauma related-injuries were mostly identified in hares (66.66%; *p* < 0.01), wild animals (88.88%; *p* < 0.001), males (75%; *p* < 0.001) during the spring–summer period (75%; *p* < 0.001), without differences in detection frequency between age ranges (*p* = 0. 461). The animals were found dead. There were generalized bone fractures (mainly spinal fractures) with surrounding hemorrhage, hemothorax or hemoperitoneum, and internal organ hemorrhages.

#### 3.1.9. Congenital Diseases

Within this category, the only condition identified was congenital glaucoma. This disorder was identified in young animals, both farm and pet rabbits (*p* = 0.102), without differences in detection between sexes (*p* = 0.581). In three animals this condition was bilateral. Reported clinical signs were blindness, weight loss and absence of pupillary and palpebral reflexes. Gross findings were buphthalmia and corneal opacity (Figure S16). Histologically, there was absence or underdevelopment of the filtration iridocorneal angles and aqueous humor outflow channels (goniodysgenesis), retinal atrophy and degeneration and corneal edema.

### 3.2. Cases with more than One Condition

In 58 animals (17.84%) of the study, more than one condition or disease was identified per animal (see Table 3). The lesions and clinical signs were similar to those described in each condition separately. The RHD along with enteritis-diarrhea disorders (*n* = 16; 27.58%) was mainly found in young rabbits (87.5%; *p* < 0.001), females (68.75%; *p* < 0.01), both farm and wild animals (*p* = 0.058) and always observed during the spring–summer period. Enteritis was jointly caused by *E.coli* an *Eimeria* spp. The presence of generalized edema along with hepatic and renal lesions (*n* = 15; 25.86%) was always identified in adult female farm rabbits during the autumn–winter period. 

These animals showed weakness and weight loss. Gross lesion included generalized subcutaneous non-inflammatory edema, more intense in the hind limbs, severe ascites, hydropericardium and hydrothorax (Figure S17). Histologically, there was multifocal interstitial nephritis, nephrosis, membranous glomerulonephritis, centrilobular to diffuse hepatic necrosis and the presence of foci of gliosis and perivascular cuffs in the CNS. Epizootic rabbit enteropathy was associated with pneumonic pasteurellosis (*n* = 11; 18.96%), in farm rabbits, mainly in breeding females (81.81%; *p* < 0.001) during the autumn–winter period. These animals also presented intense lymphoid depletion in the spleen. Peritonitis was associated with intestinal rupture due to cecal impaction, metritis and torsion and uterine rupture (*n* = 7; 12.06%; Figure S18). In five (8.62%) young female farm rabbits with cutaneous abscesses, the presence of spores and pseudocystsis of *E. cuniculi* were observed in the CNS and kidney, although no clinical symptoms were noted. Finally, four (6.89%) young female farm rabbits with myxomatosis had exudative pneumonia caused by *P. multocida* along with the presence of spores and pseudocysts in the CNS consistent with *E. cuniculi*, unrelated to nervous symptoms.

## 4. Discussion

The type, frequency and pathological features of the conditions identified in this work were in agreement with the most important lagomorph diseases already reported in other European countries as well as in other referenced studies [10,17,18]. In our case, the data must be interpreted in the light of their retrospective nature. Studied animals were submitted to the laboratory randomly over time, so there is a bias in terms of origin or species, the most commonly examined animals being farm rabbits. 

Parasitic diseases were the conditions most frequently detected, particularly those causing digestive disorders. Clinical disease was observed mostly in young animals affected by intestinal and hepatic coccidiosis or encephalitozoonosis, coming from traditionally managed rabbit farms. Factors such as poor hygienic sanitary conditions, inadequate management systems and poor health prophylactic protocols still present in this type of farms, together with immature immune systems in young animals, probably promoted those disease outbreaks [19,20]. In addition, there was a marked seasonal pattern (mainly during the autumn–winter period), with favorable environmental conditions for the resistance and transmission of parasitic agents [21] (pp. 343–356). Encephalitozoonosis was the most frequent parasitic disorder, and the second most common condition, of the study. The relevance of this disease acquires importance not only froma productive or animal welfare point of view, but also from its zoonotic potential [22]. Most of the affected animals showed the subclinical disease and only a small number were reported to show the evident clinical symptoms that are usually associated with large parasitic loads [23]. All the rabbits in which this disease was diagnosed were euthanized and no direct deaths were recorded. Altogether, these findings would suggest that these animals were well adapted to this microsporidian agent [23]. A significant number of rabbits in which encephalitozoonosis was diagnosed were simultaneously affected by other diseases such as myxomatosis, pneumonic pasteurellosis or skin abscesses, or were highly parasitized. As described in humans, it seems feasible that immunosuppressive conditions might have encouraged infection and the clinical picture [24,25]. In the cases of intestinal coccidiosis, the severity of the clinical and pathological findings made highly pathogenic species such as *E. magna* and *E. intestinalis* the most likely etiology. A significant number of animals with intestinal coccidiosis showed concomitant infections with *E.coli* and *Clostridium* spp. The so-called “rabbit enteritis complex” was the fourth most frequently diagnosed condition and the second disease responsible for digestive disorders. Imbalances in intestinal physiology associated with nutritional disorders or by primary infections caused by some of these agents are responsible for these concomitant infections [26]. In contrast, hepatoperitoneal cysticercosis, parasitic gastritis or mite infestations were significantly related to the adult age class and always seen in wild animals. The mild clinical signs found suggest a tendency towards a host–parasite equilibrium. The absence of hepatoperitoneal cysticercosis in farm animals found in this study has been associated with the needs of the parasite’s evolutionary cycle [27]. *Graphidium* spp. parasitosis is also influenced by host sex, since males were the most affected individuals, similarly to other wild populations [28], without a clear explanation of this bias. High parasitic loads are associated with concomitant infections such as myxomatosis or RHD, which in this case were not observed. Although not investigated in the present study, the number of parturitions in female rabbits or the length of breast feeding, directly related to the individual physiological state, have been seen to influence the prevalence of psoroptic mange on farms [29].

Bacterial diseases showed a presentation pattern similar to parasitic conditions, except that they were more prevalent among breeding females from industrialized farms. Bacterial mastitis, skin abscesses, pneumonic pasteurellosis and septicemic processes showed similar epidemiological characteristics. Bacterial mastitis was the most frequent female reproductive system disease in our study, followed by neoplastic processes and uterine rupture and torsions. Its incidence increases with age and the number of births [30,31] (pp. 183–193). Similar etiologic agents were identified in these four conditions, all of them considered to be opportunistic agents that need other factors such as previous trauma to the nipples or skin, climatic stress (with a marked seasonal autumn–winter pattern), or overcrowding in the case of intensive breeding to trigger the onset of all these diseases [13,32] (pp. 337–369). Skin abscesses were the most frequent dermal condition in farm rabbits, mainly associated with *Staphylococcus* spp. and directly related to the presentation of generalized pyogenic septicemic processes [33]. On the other hand, pseudotuberculosis and tularemia are rare diseases in farm animals while frequently diagnosed in wild animals, as in this study. The fact that examined cases appeared in subclinically infected adult animals could indicate some resistance to this disease and the need of concomitant factors for the occurrence of epizootic outbreaks of high mortality [34]. Although in this study both diseases appeared more frequently in females, no references were found regarding sex predilection. Tularemia was observed only in wild hares, with a greater majority of cases in the spring–summer period, probably linked to favorable weather conditions for maintenance of the transmission vectors of this infection, as observed by the high tick infestations the affected animals presented [35]. The absence of diagnosis of tularemia in wild rabbits from the same areas, where they share the same environment with affected hares, could indicate some resistance in this species. Taking into account that the clinical and pathological findings are very close to other diseases such as Tyzzer’s disease, listeriosis and pseudotuberculosis, there is a need for diagnostic confirmation by microbiological culture. The importance of this disease also lies in its potential zoonotic role [36]. 

Epizootic rabbit enteropathy was the most frequently diagnosed condition of the study. The disease occurred as epizootic outbreaks within farms with a subacute presentation. Although this condition can occur in animals of any age, in our case, it was detected mainly in young individuals. In accordance with our results, it has been reported that the season of the year is directly related to this syndrome, with a greater number of cases in autumn–winter period due to increases in food intake [37]. The pathogenesis of this disease is still unclear, although it is thought that it may have an infectious origin since the outbreaks are controlled with antibiotics. However, the main hypothesis is that any factor that favors intestinal dysbiosis may be behind the problem [38]. Animals with this disease also presented pneumonic pasteurellosis, as a reflection of the opportunistic nature of this condition, which can exacerbate the digestive disorders.

Hepatic steatosis observed in young non-obese animals was linked to prolonged fasting due to poor management or stressors. However, the presence of this condition in pregnant obese rabbits with pregnancy toxemia was associated with a negative energy balance in the final phase of pregnancy, and where a certain seasonal trend has been seen, mostly in the winter–autumn period [39,40]. The dystrophic calcifications observed in kidney and elastic arteries are associated with vitamin supplements rich in vitamin D, calcium and phosphorus and where age is usually a risk factor [41]. Although mineralization in arteries is common, cartilaginous and bone metaplasia with bone marrow formation is rare and is usually associated with previous injuries (e.g., atherosclerosis), which in this case were not detected [42].

RHD was the third most common condition detected in this study and, together with myxomatosis, the only viral disease. Currently, both disorders are thought to affect mainly wild species since the establishment of effective vaccination programs has greatly reduced their presence in industrialized farms. The cases of RHD remitted showed a marked seasonal pattern since they were more numerous both in spring and autumn, coinciding with the epizootic outbreaks of the disease in the wild, and with young and female animals as the most susceptible [43,44]. Hares affected by myxomatosis were not submitted during the last year, despite the enormous prevalence of this disease in southern Spain, which indicates that this disease has not already seriously affected the hare populations of northern Spain [45]. The intense lymphoid depletion and lymphocytolysis observed in both diseases may be responsible for the intercurrent enteric and pneumonic disorders detected in these animals [46].

The most frequently found cases of toxicoses were associated with the overdose of anticoccidiostatic drugs, mainly monensin and salinomycin, carried out in animals from traditional farms where abusive treatments with these drugs have caused parasitic resistance resulting in higher dosages and causing toxicity problems [47,48]. The cases of severe generalized edemas found in some animals in this study were always related to significant liver and kidney degenerative lesions, probably as a result of the damage caused by the use of therapeutic substances. 

Neoplasms affected exclusively adult pet rabbits. The increased life expectancy of these animals allows the development of these disorders that are not evidenced in farm animals, or may go unnoticed. The detection frequency and type of tumors were similar to other studies [10,49]. 

Other less frequently detected conditions were deaths caused by heat stroke, traumas and congenital glaucoma. Lagomorphs, mainly young or pregnant animals, are very sensitive to thermal stress. Above 35 °C, these animals can have difficulty discharging excess heat [50]. The non-specificity of lesions found makes it necessary to the differential diagnosis with other processes such as RHD, peracute myxomatosis, other peracute septicemia or toxicoses. Rabbits and hares have a long, fragile skeletal systems compared to their heavy musculature and are prone to suffer bone fractures. In this case, most skeletal traumas were associated with road accidents. Finally, glaucoma in rabbits is typically a congenital inherited condition. In our study, cases were detected both in farm and pet rabbits. In farms, animals carrying the defective gene are usually slaughtered since they have small litter sizes and decreased neonatal survival rates [51].

## 5. Conclusions

The results of this study provided information on several aspects of the main diseases affecting lagomorphs. Additionally, they also emphasized the relevance and utility of pathological diagnostic methods, both gross and histological, since, in combination with some ancillary tests, they were able to reach a definitive diagnosis in the vast majority of the submitted cases. This work also showed that lagomorph species had the same general susceptibility to diseases as other intensively farmed or domestic animals, with similar mortality risks. These disorders, although they were also present in industrial farms, were accentuated in rabbits managed under traditional farm systems, with no strict health controls or prophylactic protocols. The sex and age of the animals, as well as the time of the year, should be considered risk factors in the presentation of the described disorders. It is remarkable that 44.30% of the conditions diagnosed in domestic lagomorphs were related to metabolic or digestive disorders, triggered by management factors, so producers should be advised in order to minimize the predisposing situations. This is supported by the fact that these conditions were not diagnosed in wild lagomorphs. In 93.65% of the wild species of the study, the predominant diseases were associated with viral and bacterial infections in accordance with what has been traditionally described in wild populations of lagomorphs. This is of great importance due the role of these species in natural ecosystems.

## Figures and Tables

**Table 1:** Overall traits of the 325 lagomorphs necropsied in the Department of Animal Health (Faculty of Veterinary Science, University of León, Spain) during 2000–2018. Distributions of animals used in this study.

Species	Age Category		Sex		Origin			Time of Year ^1^	
	Young	Adult	Female	Male	Farm	Pet	Wild	S–S	A–W
Rabbits (*n* = 296)	173	123	212	84	228	34	34	109	187
Hares (*n* = 29)	8	21	22	7	-	-	29	13	16
Total (*n* =325)	181	144	234	91	228	34	63	122	203

^1^ S–S: Spring–Summer; A–W; Autumn–Winter.

**Table 2:** Summary of diseases or conditions diagnosed as a single cause of death or illness in lagomorphs referred to Department of Animal Health (Faculty of Veterinary Science, University of León, Spain) during 2000–2018.

Conditions	*n* (%)	Species		Age Category		Sex		Time of Year		Origin		
		Rabbit	Hare	Young	Adult	Female	Male	S–S	A–W	Farm	Pet	Wild
Acute shock (Heat stroke)	5 (1.87)	5	-	5	-	-	5	5	-	-	5	-
Arterial bone metaplasia	4 (1.49)	4	-	-	4	3	1	4	-	2	2	-
Blue breast	10 (3.74)	10	-	1	9	10	-	3	7	10	-	-
Congenital glaucoma	4 (1.49)	4	-	4	-	2	2	3	1	2	2	-
Ectoparasites (mite)	4 (1.49)	4	-	-	4	4	-	3	1	4	-	-
Encephalitozoonosis	25 (9.36)	25	-	16	9	16	9	6	19	19	6	-
Hepatic coccidiosis	13 (4.86)	13	-	11	2	6	7	8	5	13	-	-
Hepatic steatosis	16 (5.99)	16	-	12	4	14	2	2	14	16	-	-
Hepatoperitoneal cysticercosis	9 (3.37)	3	6	2	7	6	3	1	8	2	-	7
Intestinal coccidiosis	7 (2.62)	7	-	7	-	4	3	-	7	7	-	-
Epizootic rabbit enteropathy	27 (10.11)	27	-	21	6	22	5	8	19	26	1	-
Myxomatosis	9 (3.37)	9	-	6	3	6	3	2	7	7	-	2
Neoplasms	12 (4.49)	12	-	-	12	9	3	8	4	1	11	-
Nephrosis ^a^	6 (2.24)	6	-	5	1	4	2	2	4	5	1	-
Parasitic gastritis	7 (2.62)	7	-	-	7	-	7	-	7	-	-	7
Pneumonic pasteurellosis	9 (3.37)	9	-	8	1	8	1	1	8	8	1	-
Pregnancy toxemia	5 (1.87)	5	-	-	5	5	-	-	5	5	-	-
Pseudotuberculosis	10 (3.74)	4	6	3	7	9	1	2	8	2	-	8
Rabbit enteritis complex	17 (6.36)	16	1	15	2	10	7	3	14	13	2	2
Skin abscesses	10 (3.74)	10	-	8	2	7	3	1	9	10	-	-
Staphylococcal septicemia	8 (2.99)	8	-	3	5	6	2	1	7	8	-	-
Toxicoses (ionophores in feed)	11 (4.11)	11	-	8	3	11	-	3	8	11	-	-
Traumatism	9 (3.37)	3	6	4	5	2	6	6	2	1	-	8
Tularemia	9 (3.37)	-	9	-	9	8	1	8	1	-	-	9
Rabbit hemorrhagic disease	22 (8.23)	21	1	14	8	13	9	16	6	9	-	13
Total	267	238	29	152	115	185	82	96	171	180	31	56

^a^ Associated with intoxications and nephrocalcinosis. Renal disorders associated with staphylococcal and *Encephalitozoon cuniculi* infections were excluded.

**Table 3:** Distribution of the number of cases in which more than one disease/condition was diagnosed in lagomorphs referred to Department of Animal Health (Faculty of Veterinary Science, University of León, Spain) during 2000–2018.

Conditions	*n* (%)	Species		Age Category		Sex		Time of Year		Origin		
		Rabbit	Hare	Young	Adult	Female	Male	S–S	A–W	Farm	Pet	Wild
Encephalitozoonosis + skin abscesses	5 (8.62)	5	-	5	-	5	-	-	5	5	-	-
Generalized edemas + Hepatic and renal dystrophies	15 (25.86)	15	-	-	15	15	-	-	15	15	-	-
Myxomatosis + Pasteurellosis + Encephalitozoonosis	4 (6.89)	4	-	4	-	4	-	4	-	4	-	.
Epizootic rabbit enteropathy + Pasteurellosis	11(18.96)	11	-	1	10	9	2	2	9	11	-	-
Peritonitis ^a^	7 (12.06)	7	-	5	2	5	2	4	3	4	3	-
Rabbit hemorrhagic disease + enteritis-diarrhea ^b^	16 (27.58)	16	-	14	2	11	5	16	-	9	-	7
Global	58	58	-	29	29	49	9	26	32	48	3	7

^a^ Associated with intestinal rupture due to cecal impaction, metritis and torsion and uterine rupture. ^b^ Bacterial and/or parasitic enteritis.

## Supplementary Materials

The following are available online at https://www.mdpi.com/2076-2615/10/1/158/s1, Figure S1: Presence of focal, irregular and depressed pale areas on the renal cortical surface in rabbits affected by *Encephalitozoon cuniculi*, Figure S2: Multifocal to coalescing irregularly shaped, raised, yellow-white nodules or cords in liver of farm rabbits affected by *Eimeria stiedae*, Figure S3: Presence of parasitic cysts in liver surface, peritoneal, diaphragmatic and intestinal serosa in a hare affected by *Cysticercus pisiformis*, Figure S4: Adult red worms of *Graphidium* genus located in gastric mucosa of wild rabbits, Figure S5: Miliary necrotic foci in the intestine of a wild rabbit infected by *Yersinia enterocolitica*, Figure S6: Multiple white-yellowish military necrotic foci in liver(a) and spleen (b) in a wild hare suffering tularemia, Figure S7: Fibrinosuppurtive pleuropneumonia associated with *Pasteurella multocida* infection in a farm rabbit, Figure S8: Microabscesses in the lung (a) and skeletal muscle (b) in farm rabbit with staphylococcal septicemia, Figure S9: Abundant translucent and gelatinous mucus in ileum and impacted cecal content in a farm rabbit affected by Epizootic rabbit enteropathy, Figure S10: Wild rabbit affected by rabbit hemorrhagic disease; (a) serosanguinolent discharge in nose and mouth; Pulmonary (b) and epicardial hemorrhages (c); (d) discolored liver with enhanced lobular pattern, Figure S11: Wild and farm rabbits affected by myxomatosis. (a) Serous blepharoconjunctivitis and (b) pseudo-tumors along periorbital area, Figure S12: Farm rabbit affected by "rabbit enteritis complex". (a) Yellowish-watery diarrhea; (b) severe intestinal dilation, catarrhal enteritis and dysbiosis caused by *Eimeria* spp. and *Escherichia coli*, Figure S13; Pet rabbit with arterial bone metaplasia. (a) Aortic arteries with a rigid and whitish appearance; (b) foci of cartilaginous and bone metaplasia with bone marrow formation in aortic arteries of a pet rabbit, Figure S14; gross appearance of the uterus of a pet rabbit with uterine adenocarcinoma, Figure S15; discoloration and degeneration of myocardial muscle in a farm rabbit poisoned with monensin, Figure S16; corneal opacity and mild buphthalmia in a farm rabbit affected by unilateral congenital glaucoma; Figure S17; generalized subcutaneous non-inflammatory edema (a) and severe ascites (b) in a farm rabbit with renal and hepatic injury, Figure S18; Fibrinous peritonitis associated with intestinal rupture due to cecal impaction in a farm rabbit.
