# Supplementary material for: Causes of Mortality and Disease in Rabbits and Hares: A Retrospective Study
Source: Animals (Basel). 2020 Jan 17;10(1):158. doi: 10.3390/ani10010158 (PMC7022519; doi:10.3390/ani10010158)
Supplement: Supplementary file 1 [file animals-10-00158-s001.pdf]

animals

MDPI

Supplementary figures:

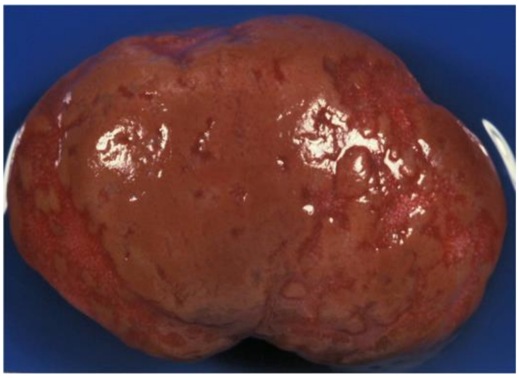

Figure S1. Presence of focal, irregular and depressed pale areas on the renal cortical surface in rabbits affected by *Encephalitozoon cuniculi.*

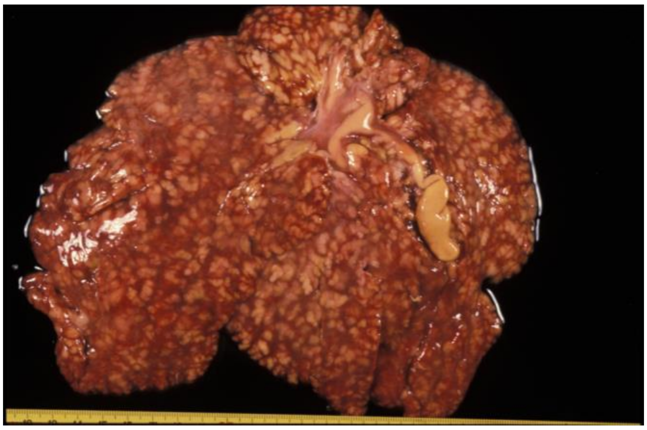

Figure S2. Multifocal to coalescing irregularly shaped, raised, yellow-white nodules or cords in liver of farm rabbits affected by *Eimeria stiedae*.

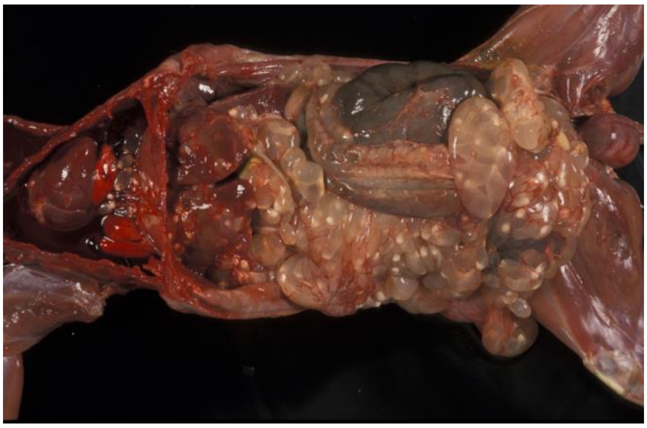

Figure S3. Presence of parasitic cysts in liver surface, peritoneal, diaphragmatic and intestinal serosa in a hare affected by *Cysticercus pisiformis*.

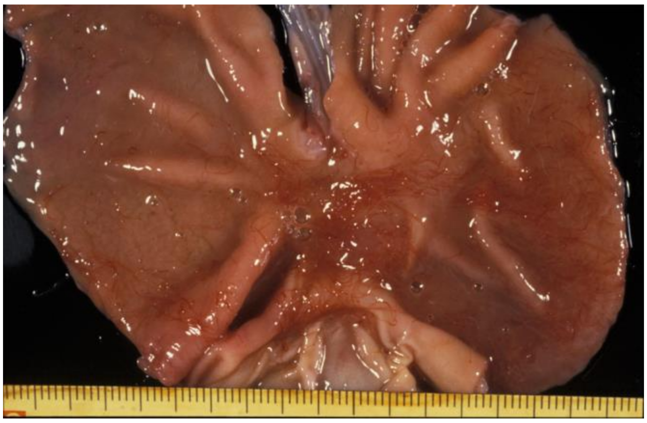

Figure S4. Adult red worms of *Graphidium* genus located in gastric mucosa of wild rabbits.

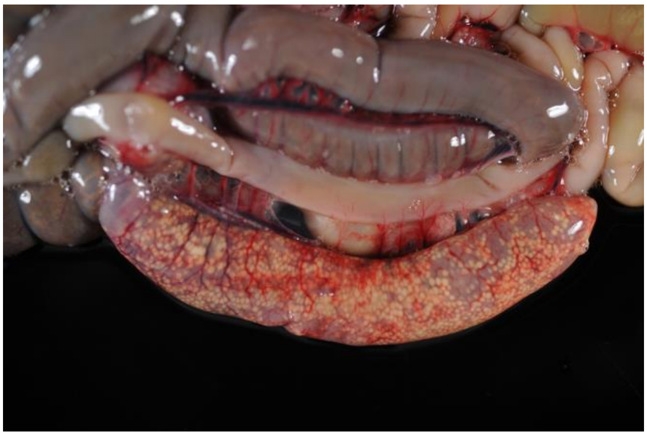

Figure S5. Miliary necrotic foci in the intestine of a wild rabbit infected by *Yersinia enterocolitica.*

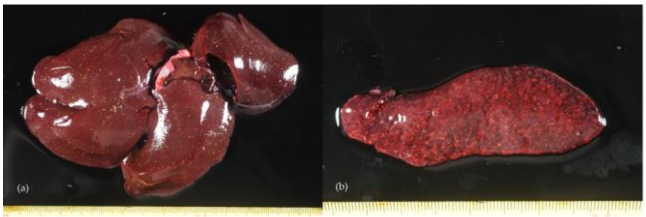

Figure S6. Multiple white-yellowish military necrotic foci in liver(a) and spleen (b) in a wild hare suffering tularemia.

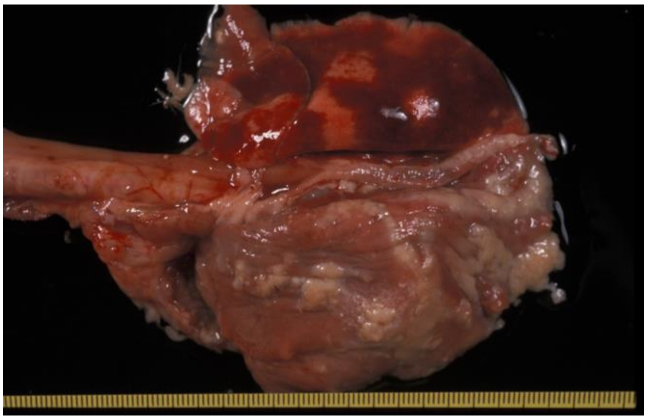

Figure S7. Fibrinosuppurtive pleuropneumonia associated with *Pasteurella multocida* infection in a farm rabbit.

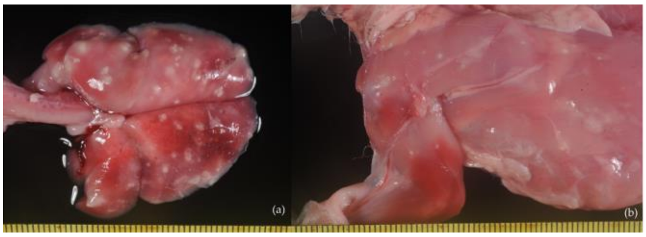

Figure S8. Microabscesses in the lung (a) and skeletal muscle (b) in farm rabbit with staphylococcal septicemia.

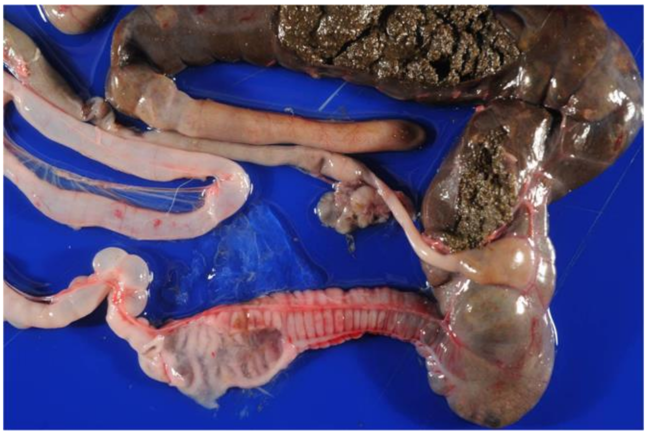

Figure S9. Abundant translucent and gelatinous mucus in ileum and impacted cecal content in a farm rabbit affected by Epizootic rabbit enteropathy.

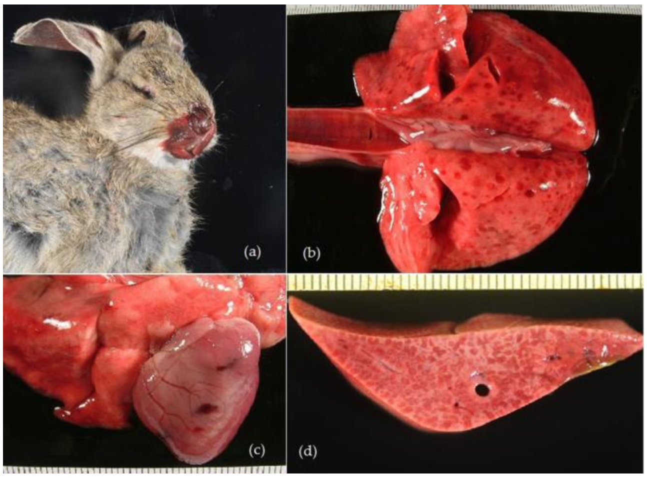

Figure S10. Wild rabbit affected by rabbit hemorrhagic disease; (a) serosanguinolent discharge in nose and mouth; Pulmonary (b) and epicardial hemorrhages (c); (d) discolored liver with enhanced lobular pattern.

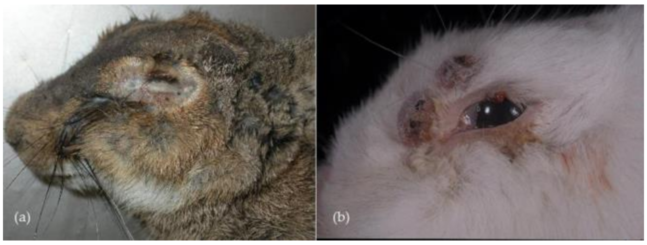

Figure S11. Wild and farm rabbits affected by myxomatosis. (a) Serous blepharoconjunctivitis and (b) pseudo-tumors along periorbital area.

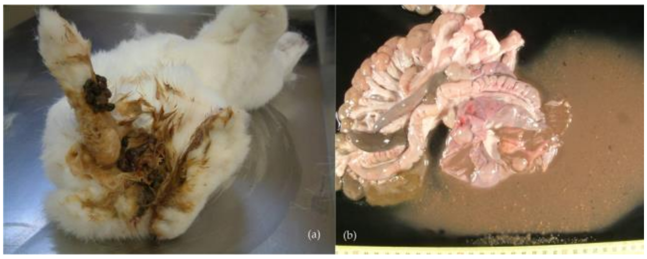

Figure S12. Farm rabbit affected by "rabbit enteritis complex". (a) Yellowish-watery diarrhea; (b) severe intestinal dilation, catarrhal enteritis and dysbiosis caused by *Eimeria* spp and *Escherichia coli*.

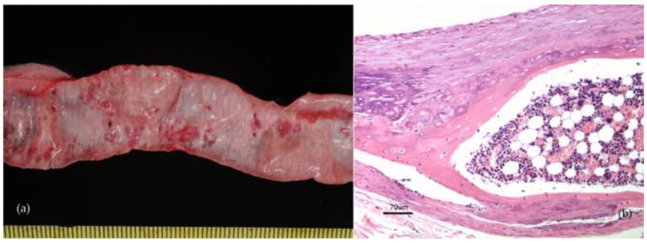

Figure S13. Pet rabbit with arterial bone metaplasia. (a) Aortic arteries with a rigid and whitish appearance; (b) foci of cartilaginous and bone metaplasia with bone marrow formation in aortic arteries of a pet rabbit.

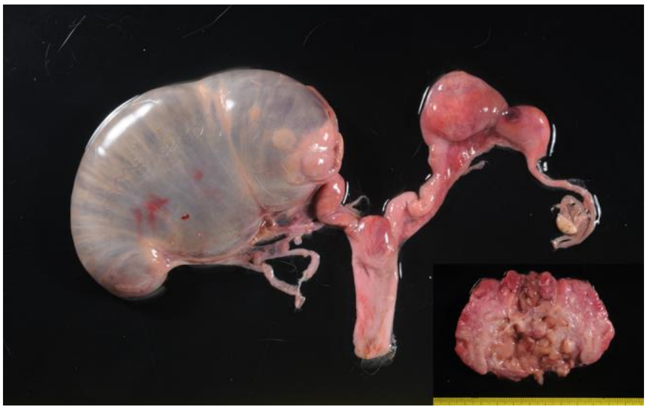

Figure S14. gross appearance of the uterus of a pet rabbit with uterine adenocarcinoma.

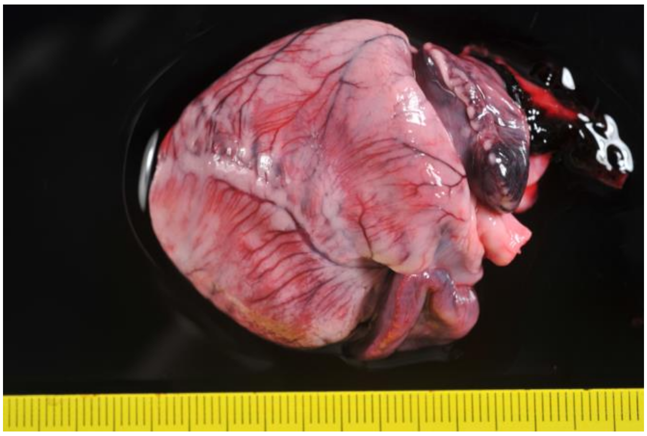

Figure S15. discoloration and degeneration of myocardial muscle in a farm rabbit poisoned with monensin.

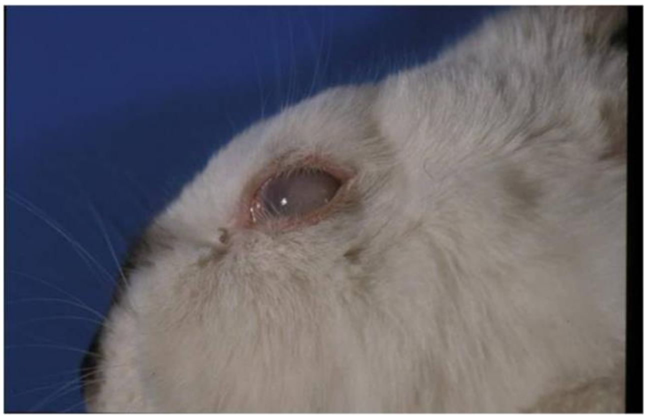

Figure S16. corneal opacity and mild buphthalmia in a farm rabbit affected by unilateral congenital glaucoma.

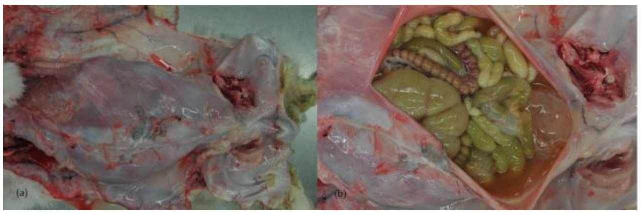

Figure S17. generalized subcutaneous non-inflammatory edema (a) and severe ascites (b) in a farm rabbit with renal and hepatic injury.

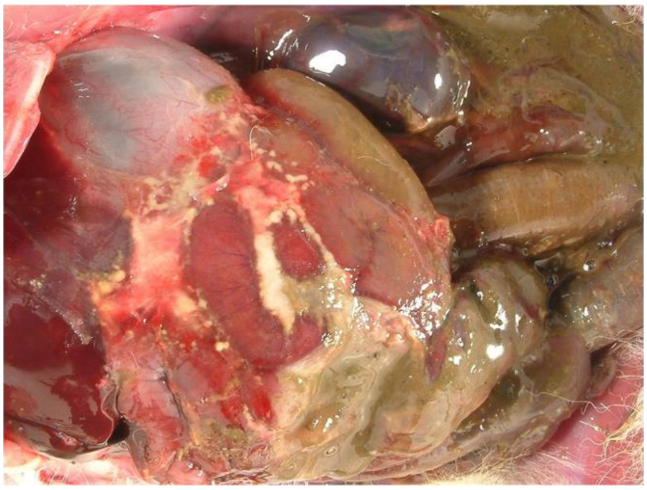

Figure S18. Fibrinous peritonitis associated with intestinal rupture due to cecal impaction in a farm rabbit.

© 2020 by the authors. Submitted for possible open access publication under the terms and conditions of the Creative Commons Attribution (CC BY) license (http://creativecommons.org/licenses/by/4.0/).
